# Supplementary material for: DMRforPairs: identifying Differentially Methylated Regions between unique samples using array based methylation profiles
Source: BMC Bioinformatics. 2014 May 15;15:141. doi: 10.1186/1471-2105-15-141 (PMC4046028; doi:10.1186/1471-2105-15-141)
Supplement: Additional file 2 — DMRforPairs output for the comparison of A431-MCF7 and NA17018-NA17105. Please start from the HTML files in each folder. Available via the BMC Bioinformatics website. [file 1471-2105-15-141-S2.zip › 1394847754114233_MOESM2_ESM/A431_MCF7/figures/7092.pdf]

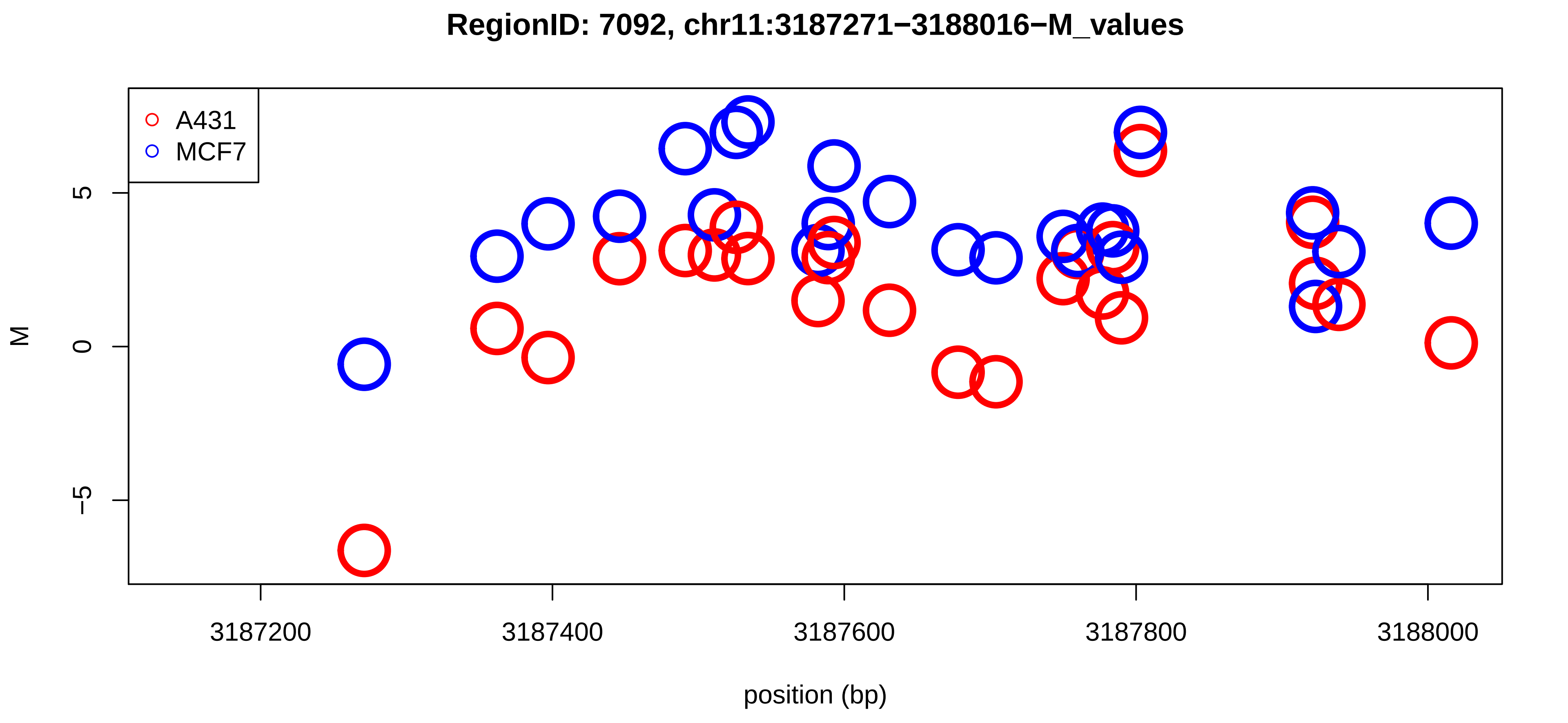
RegionID: 7092, chr11:3187271−3188016−M_values
A431
MCF7
M
−5
0
5
3187200
3187400
3187600
3187800
3188000
position (bp)

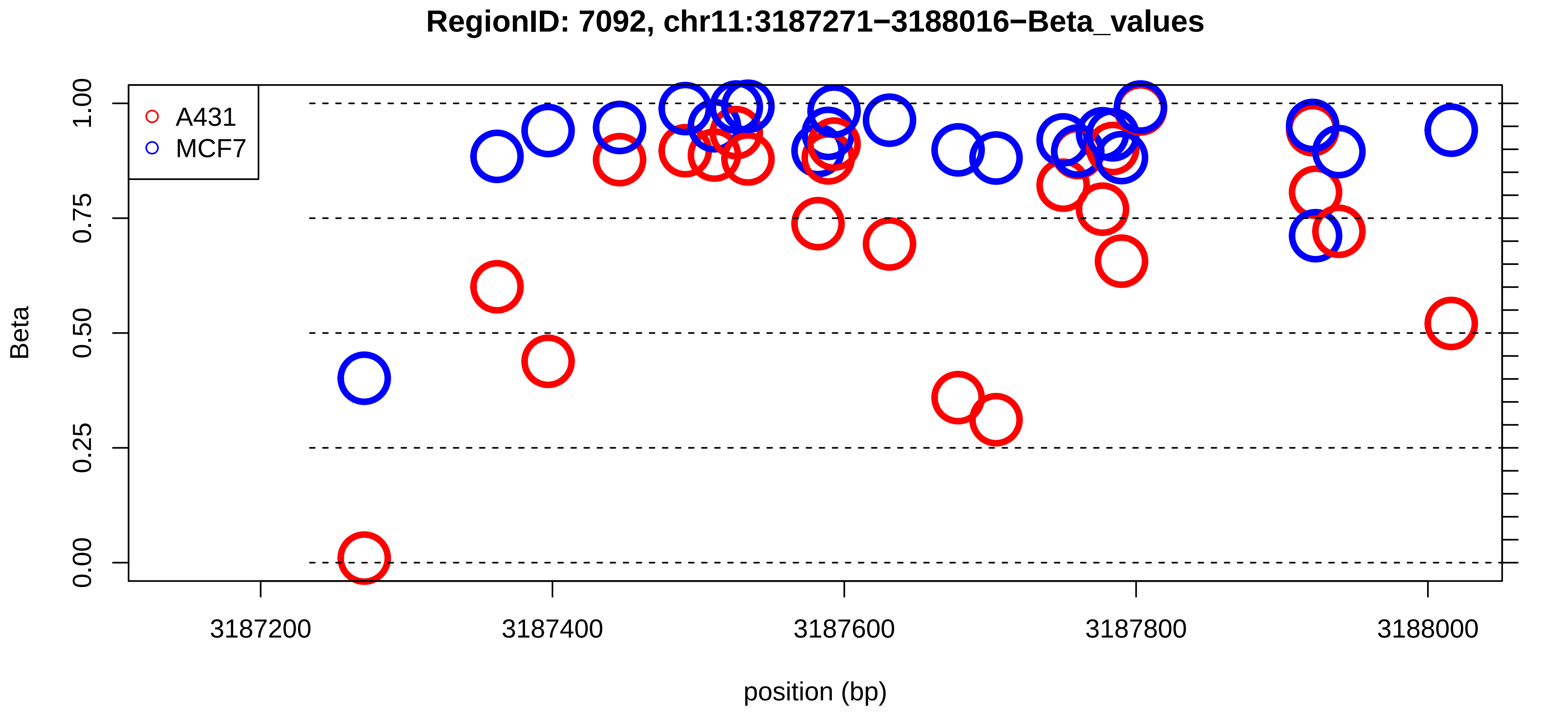
RegionID: 7092, chr11:3187271−3188016−Beta_values
A431
MCF7
Beta
0.00
0.25
0.50
0.75
1.00
3187200
3187400
3187600
3187800
3188000
position (bp)

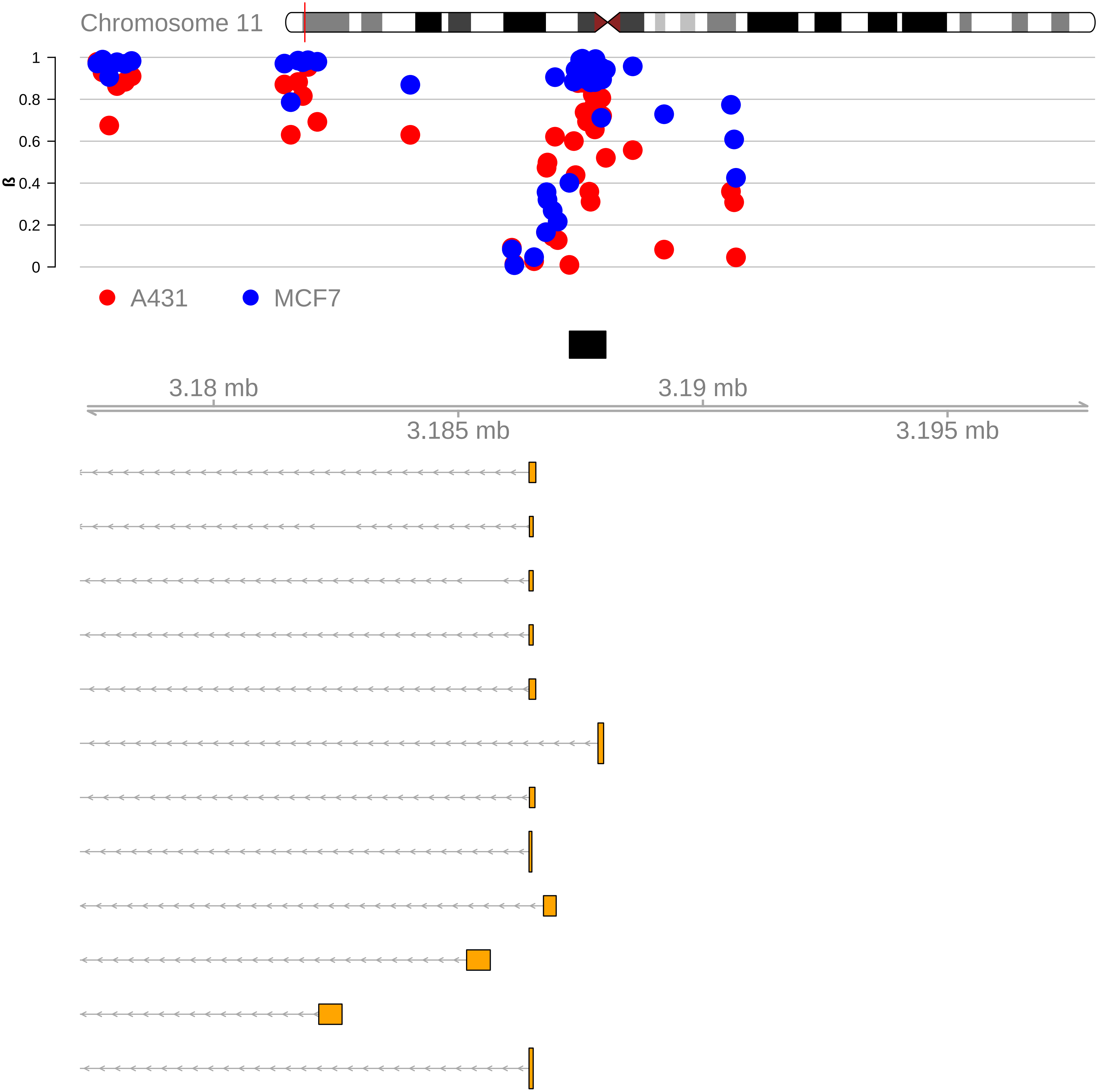

Chromosome 11
1
0.8
0.6
β 0.4
0.2
0
A431
MCF7
3.18 mb
3.19 mb
3.185 mb
3.195 mb
